# Evaluating an Oral Health Education Intervention in Chinese Undocumented Migrant Mothers of Infants in Northern Ireland

**DOI:** 10.3390/dj7010008

**Published:** 2019-01-19

**Authors:** Siyang Yuan

**Affiliations:** Dental Health Services Research Unit, School of Dentistry, University of Dundee, Park Place, Dundee DD1 4HN, UK; s.z.yuan@dundee.ac.uk

**Keywords:** undocumented migrant, baby oral health, oral health education, parental knowledge, attitudes and behaviors

## Abstract

Background: Poor oral health remains a significant dental public health challenge for ethnic minority and immigrant groups living in the UK. This study aimed to evaluate a culturally appropriate community-based home visiting oral health education intervention for Chinese, undocumented migrant mothers to promote their infants’ oral health, by focusing on their oral health related knowledge, attitudes, and behaviors. Methods: A convenience sample of 36 Chinese mothers with babies aged less than eight weeks were recruited in South-East region of Belfast. The local Chinese community was consulted to assist with the development of the intervention. The oral health education intervention was provided to 19 intervention group mothers through home visits and telephone calls during mothers’ first postpartum year. They were also provided with unlimited social support during the intervention period. Mothers’ oral health related knowledge, attitudes, and behaviors regarding baby toothbrushing and sugar snacking were measured at eight weeks, six months, and 12 months. Results: A higher proportion of Chinese intervention group mothers had improved knowledge about baby toothbrushing at 12 months compared with control group mothers (χ^2^ = 14.12: *p* = 0.004). Significantly, more intervention group mothers’ oral health related attitudes were enhanced regarding baby toothbrushing and sugar snacking compared with control group mothers. Conclusion: This community-based oral health education intervention has shown effects in mothers’ self-reported knowledge, attitudes, and behaviors in the intervention group when the community based and culturally appropriate home-visiting program improved the mothers’ oral health related knowledge, attitudes, and behaviors.

## 1. Introduction

Untreated dental caries has been shown to be the greatest disease burden in the world, with untreated caries of deciduous teeth ranked as the 10th greatest global disease burden [1]. As a consequence, children suffer poor oral health and poor quality of life, especially for those from socially disadvantaged backgrounds [2].

The association between socioeconomic status (SES) and health outcomes has been well established and it further affected by such factors as ethnicity [3,4]. For decades, research on ethnic disparities in health has provided evidence that health inequalities may decrease or even disappear if SES inequalities were eliminated [5]. However, in recent years, the focus on racial/ethnic health inequalities has shifted from “race or class” to the intersectionality between race/ethnicity and class [6]. For example, findings from a longitudinal study of a birth cohort showed that health gains from improved family economic resources were smaller for Black than for White young people [7]. Similarly the effects of parental education on families’ functioning to escape poverty was larger for White compared to Black [8]. Based on the ‘Diminished Return theory for Minorities’, race/ethnicity and SES are two different and interrelating factors of social position that may explain the role of racial/ethnic upon health inequities [6,9].

During the migration process, immigrant families face multiple disadvantages when adapting to the host society in terms of lifestyle and cultural norms. These disadvantages include financial hardship for settlement, language difficulty, little knowledge of available social, and healthcare resources and psychosocial stress caused by their unsettled immigration status. With regard to oral health, migrant children have been shown to have poorer oral health outcomes compared with children in the host country [10]. It has been suggested that this may be due to the fact that migrant childhood is exposed to multiple disadvantages during the migration process, which had adverse consequences for growth and health. As possible consequences of these socioeconomic and cultural disadvantages, children from newly arrived immigrant families are more likely to suffer the poorest oral health. A recent Australian study showed that length of time as a resident in the host country was an independent predictor for the preschool children’s obvious caries experience [11].

An additional factor in a child’s obvious decay experience is the association between maternal oral health related knowledge, beliefs and practices, and child oral health [12,13,14,15]. Furthermore, mother’s cariogenic bacteria can be transmitted to the child, which has been shown in the association of maternal and child’s levels of Streptococcus mutans (MS) [16]. Other studies have reported an association between children’s caries experience and their mothers’ poor oral health status [13,16]. More significantly, mothers who do not appreciate the importance of child oral health are less likely to brush their children’s teeth with fluoride toothpaste [16]. It is therefore of importance to address these factors when designing a community-based oral health promotion intervention to improve parents’ oral health related knowledge, attitudes, and behaviors regarding child oral health.

Unfortunately, little research investigating, specifically, Chinese migrant parents’ oral health related knowledge, beliefs, and practices are available. Wong et al. explored Chinese parents’ perceptions regarding oral hygiene and access to dental care. They found that parents had proper knowledge about the best feeding practice and had fatalistic attitudes toward child tooth decay [17]. The lack of oral health related knowledge and positive attitudes created barriers for Chinese parents when they sought preventive dental care for their young children. Wong et al.’s findings further underscore the need of an oral health promotion intervention that is culturally appropriate for Chinese migrant parents [17]. 

Community-based interventions, such as home visiting programs, have been demonstrated to be effective in raising parental awareness of, and assisting them to, adopt healthy lifestyle behaviors that are beneficial to children’s health needs [18,19,20]. Using a one-to-one health educational approach, community health workers who are from similar ethnic and social backgrounds will be more likely to understand the psycho-social difficulties, concerns, and health needs of newly arrived immigrant families. This, as proposed, will build up trusting relationships between parents and oral health professionals and will enable the delivery of oral health messages. 

The aim of this study therefore was to examine the effect of a community-based, home visiting oral health education intervention delivered in a culturally appropriate approach for Chinese newly arrived undocumented migrant mothers. It focused on promoting mothers’ oral health related knowledge, their intentions to brush their babies’ teeth with fluoride toothpaste and to control baby sugar snacking, their oral health related behaviors as well as their own oral health in terms of obvious decayed experience.

## 2. Materials and Methods

### 2.1. Study Design and Setting

The study design was a quasi-experimental study with no randomization of the participating Chinese newly arrived undocumented migrant mothers. Undocumented migrants are people who are living without a valid residence permit authorizing them to permanently stay in the country in which they are currently residing [21]. The study setting was in the South and West regions of Belfast in the UK. In Northern Ireland (NI), Chinese migrant group constitutes the largest ethnic group with an approximately size of population of 8000 [22]. The majority of the Chinese population originate from Hong Kong and have been residents in NI since the 1960s. However in recent years, Chinese people from Northern region of Fu Jian province of Southern China, one of the most deprived areas in China, have migrated to NI. Most of the Fu Jian men work in catering services for long hours with low pay. The Fu Jian undocumented migrant mothers are unable to speak much English and remain at home most of the time.

In terms of the dental health care system in Northern Ireland, people have to pay for their dental treatments unless they are entitled to free National Health Service (NHS) dental treatments [23]. For example, it is free for children under 18 and pregnant women as well as women with babies aged up to 12 months to receive dental examinations and treatments in NHS dental care services. 

### 2.2. Sample

A sample size of 17 Chinese migrant mothers in the intervention group, and 17 in the control group, was calculated to have 80% power to detect a difference in the caries incidence of 0.56 between the intervention and control group mothers’ D_3_MFT assuming that the common standard deviation is 0.55 using a two-group t-test with 0.05 two-sided significance level.

On statistical advice, the control group mothers were recruited six months before the recruitment of intervention group mothers. This was to reduce the possibility of contamination from this oral health education intervention. The intervention group mothers were selected to match the demographic profile (i.e., mother’s age, educational attainment, level of relative deprivation, etc.) of the control group mothers. Both intervention and control group mothers have been settled in the UK for no longer than three years. All participants were undocumented migrants. A “snowball sampling” technique was used in order to recruit this ‘hard to reach’ group of newly arrived undocumented migrant mothers [24]. This strategy relied on the initial participating mothers to provide access to other members of their group or community by word of mouth.

### 2.3. Intervention Program

The intervention aimed to promote Chinese newly arrived, undocumented migrant mothers’ oral health related knowledge, their intentions to brush baby’s teeth using fluoride toothpaste and to control baby sugar consumption, and their oral health related behaviors in the first 12 months of the baby’s life. Therefore, prior to the development of the program, the Chinese Welfare Association and community leaders were contacted. The nature of the program was explained and advice requested. Using the information received from talking with Chinese migrant mothers with toddlers born in Belfast and understanding their health and social needs, the program was formulated. 

The community-based oral health education intervention was delivered by a Chinese community health worker (SY) in a culturally appropriate way through home visiting and telephone contact at five different time points over the intervention period (Table 1). During the home visits, SY provided information about oral health focusing on promoting healthy feeding (including introducing suitable foods and drinks for the benefits of baby’s teeth), baby teething, and benefit of twice daily toothbrushing using fluoride toothpaste as well as regular dental attendance for both mothers and babies. She also demonstrated the correct toothbrushing techniques to the mothers in the intervention group and encouraged them to use the teething and oral hygiene resources as well as a trainer cup. These were provided during the intervention. In addition, the trust between the Chinese health worker and mothers was built throughout the intervention period and social support provided, based on mothers’ needs such as referring them to the local social services and health and/or oral health services. 

### 2.4. Data Collection

#### 2.4.1. Questionnaire

The questionnaire was developed in several parts, which included demographic information, assessments of the mother’s oral health related knowledge, attitudes, and behaviors. Mothers’ demographics included information such as their age, marital status and postal codes of their residential areas. The post code would enable an assessment of social deprivation to be made using the Noble Index of Deprivation [22]. The Noble Index of Deprivation is a multiple deprivation measure that includes information from the seven domains of: Income, employment, health and disability, education skills and training, proximity to services, living environment, and crime and disorder. 

The attitudinal items were assessed on a five-point Likert scale ranging from “strongly disagree” (scoring 1) through “neither agree nor disagree” (scoring 3) to “strongly agree” (scoring 5). These questions were derived from previous studies and had a good reliability and validity [26]. The questionnaire contained one oral health related knowledge question with a single choice answer at the baseline assessment regarding the age of babies that mothers think they should start brushing baby’s teeth. A section was included for the six-month and 12-month questionnaire assessments to evaluate mothers’ knowledge and behaviors with regard to baby toothbrushing using fluoride toothpaste. If the mother answered ‘Yes’, then she would be asked to complete this section about baby toothbrushing; otherwise she would be advised to go to the next section of the questionnaire. Questions regarding mothers’ own oral health related behaviors such as toothbrushing behavior and dental attendance were included in the questionnaires using single choice answers such as “how many times do you brush your teeth during the day?” and “what is your usual reason for going to see a dentist?”.

#### 2.4.2. Assessment of Mothers’ Dental Health Status

Prior to the evaluation, an independent and calibrated dental examiner (AS) who was blind with regard to the aim of the study was invited to examine all the participants’ teeth. After training and calibration, AS’s intra-examiner reliability was 0.94 (Kappa value). 

Mother’s dental health status was assessed using obvious decay experience (D_3_MFT). The protocol used recognizes decay, which extends into the dentine on the basis of a clinical examination conducted without the use of probes [27]. Dental caries were diagnosed at the decay into dentine (D_3_) threshold using a visual method (including visual dentine caries) without radiography, fiber-optic transillumination, or compressed air. The mothers’ teeth were inspected under standardized illumination. The calibrated dental examiner (AS) used a flexi-lum light and mouth mirror. All necessary steps were taken to prevent cross-infection. For example, disposable gloves and disposable mirrors were used and collected in medical waste bags and were disposed of in hospital.

#### 2.4.3. Outcome Measures

The primary outcome measures were mothers’ oral health related knowledge, attitudes and behaviors with regard to baby toothbrushing, sugar consumption and baby tooth decay as well as maternal dental health behaviors, measured at eight weeks, six months, and 12 months. The secondary outcome measure was mothers’ obvious decay experience examined at eight weeks, six months, and 12 months.

### 2.5. Ethical Considerations

Ethical approval was granted from the Office for Research Ethics Committees Northern Ireland (Ref: 05/NIR02/64). All subjects gave their informed consent for inclusion before they participated in the study.

### 2.6. Statistical Analysis

Data from questionnaires were entered into SPSS 12.0.1. Frequencies were computed to describe the demographic profile of mothers of babies. Mother’s attitudinal questions regarding their intentions to brush baby’s teeth using fluoride toothpaste and to control baby sugar consumption were summed according to the Likert scales developed from factor analyses from the previous study [26]. Chi-squared analysis and Fisher’s exact test was used to compare mothers’ oral health related knowledge and self-reported behaviors between intervention and control groups at different time points throughout the intervention period. T-test analyses were conducted to compare differences in maternal oral health related attitudes and mothers’ oral health outcomes at each assessment between intervention and control groups. 

The differences with regard to changes over intervention time in the mean scores of maternal oral health related attitudes and their oral health outcomes between baseline (eight weeks) and 12 months were compared between intervention and control groups using t-tests. The use of differences in the mean scores allowed the analyses of all the data while excluding the two missing mothers at 12 months. 

## 3. Results

A convenience sample of 36 Chinese newly arrived undocumented migrant mothers of new babies was recruited. One mother from the intervention group and one mother from the control group were lost to the 12-month follow-up as they moved from NI. The baseline information indicated that mothers’ demographic characteristics were comparable between intervention and control group (Table 2).

### 3.1. Mothers’ Oral Health Related Knowledge

At baseline, sixteen (44%) mothers irrespective of their groups correctly answered the question that they should start brushing baby’s teeth as soon as the first teeth erupt. No other statistically significant difference was shown between intervention and control group mothers (χ^2^ = 0.45: *p* = 0.50).

At six-month assessment, of the 18 mothers who stated they had started brushing baby’s teeth, a higher proportion of intervention group mothers (100%) knew that they should start brushing baby’s teeth as soon as the first teeth erupted and the time they should start using fluoride toothpaste, compared with control group mothers (50%). However, there were no statistically significant differences between the two groups (χ^2^ = 8.47: *p* = 0.11). Sixteen intervention group mothers knew the right amount of fluoride toothpaste (i.e., “smear-size”) when brushing their baby’s teeth, as compared with control group mothers (*n* = 2). 

At 12-month assessment, of the 20 mothers who stated they had started brushing baby’s teeth, a statistically significant higher proportion of intervention group mothers (100%) knew that they should start brushing their baby’s teeth and use fluoride toothpaste as soon as the first teeth erupted, compared with control group mothers (25%) (χ^2^ = 14.12: *p* = 0.004). 

### 3.2. Mothers’ Oral Health Related Attitudes

No statistically significant differences were shown in mothers’ oral health related attitudes with regard to their intentions to brush baby’s teeth and to control baby sugar consumption between intervention and control group mothers at baseline (Table 3). 

At six-month follow up, intervention group mothers had statistically significant higher mean attitudinal scores in “importance and intention to brush baby’s teeth” and “importance and intention to control baby sugar snacking” compared with control group mothers (Table 3). Similar findings were shown in their 12-month follow-up assessment (Table 3).

**Figure 1:**
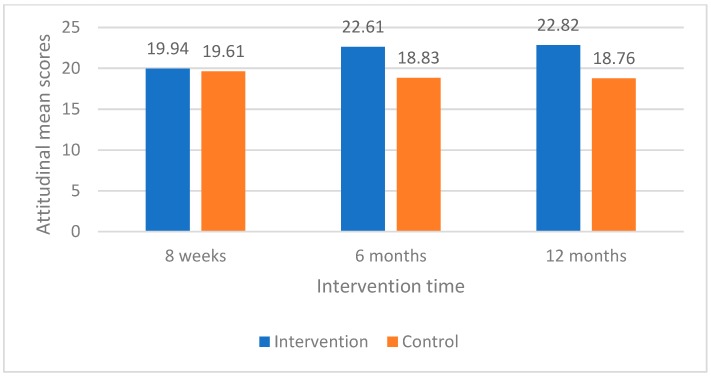
Changes in mean scores for importance and intention to brush baby’s teeth for intervention and control groups.

**Figure 2:**
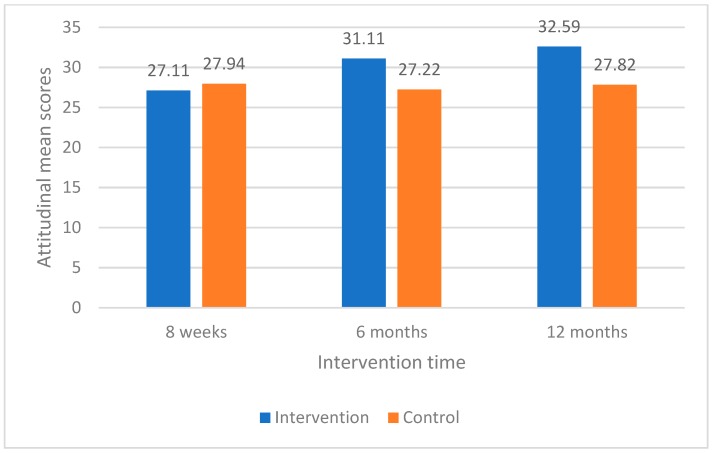
Changes in mean scores for importance and intention to control baby sugar snacking for intervention and control groups.

When the changes of mothers’ attitudinal mean scores over intervention period (eight weeks compared with 12 months) were measured, intervention group mothers had statistically significant changes in their perceived “importance and intention to brush baby’s teeth” and “importance and intention to control baby sugar snacking” compared with control group mothers (Table 4). These changes in mothers’ attitudinal mean scores between baseline (eight weeks), six months, and 12 months between intervention and control groups were also shown in line graphs (Figure 1 and Figure 2).

### 3.3. Mothers’ Oral Health Related Behaviors

At baseline, twenty-eight (78%) mothers reported brushing their own teeth using fluoride toothpaste at least twice daily. No statistically significant difference was shown regarding reported daily frequencies of toothbrushing between intervention and control group mothers (χ² = 0.64: *p* = 0.42). Twenty-three (64%) mothers stated they were registered with a dentist. Eight (22%) mothers stated that they attended for regular dental examinations and the remainder reported that they went to see a dentist either for treatment (6%) or only if having problem with their teeth or gums (47%). Nine (25%) mothers stated that they had never visited a dentist. No statistically significant differences were shown with regard to mothers’ dental attendance at baseline.

At six-month assessment, 18 mothers (50%) stated that they had started brushing their baby’s teeth at six months. Statistically significant higher proportions of intervention group mothers (89%) compared with control group mothers (11%) stated that they brushed their baby’s teeth (χ² = 21.78: *p* < 0.001). Statistically significant higher proportion of mothers from the intervention group compared with the control group stated that they brushed their own teeth at least twice a day (χ² = 5.78: *p* = 0.04).

Twenty mothers (59%) reported that they brushed their baby’s teeth at 12 months. Statistically significantly higher proportions of intervention group mothers (94%) stated that they brushed their baby’s teeth (*p* < 0.001), compared with control group mothers (24%). No other statistical differences were found in mothers’ other oral health related behaviors such as regular prevention oriented dental attendance. 

### 3.4. Mothers’ Obvious Decay Experience

There were no statistical significant differences in mothers’ obvious decay experience (D_3_MFT) between intervention and control group mothers at baseline (*t* = 0.43: *p* = 0.67), six months (*t* = 0.40: *p* = 0.69), and 12 months (*t* = 0.10: *p* = 0.92). No statistical significant difference in mothers’ obvious decay experience (D_3_MFT) over intervention period was found between intervention and control group mothers (*t* = −1.05: *p* = 0.30). 

## 4. Discussion

The intervention has shown the promising effect in improving mothers’ oral health related knowledge, perceived importance, and intention to take care of baby’s teeth in terms of baby toothbrushing and sugar snacking, and their improved self-reported baby toothbrushing behaviors. These findings have further addressed the importance of tailoring the community based oral health intervention through including the local community in the development of the intervention. More importantly, this intervention supports the proposition that health intervention for newly arrived migrant groups should be provided in a culturally appropriate manner using community health workers who speak the same language and share the same cultural background. 

The recruited Chinese undocumented migrant mothers were characteristic of those with multiple social disadvantages: being undocumented migrants, had language difficulties, experienced problems in adapting the mainstream society, had lower levels of educational attainment, and resided in disadvantaged areas [28,29]. The limited literature suggested children of migrant families had poorer oral health compared with children of indigenous families [30]. A recent American study indicated a social pattern in children’s regular dental visits with children of non-permanent residents having the lowest dental care utilization rate (32% had one or more dental visits in the last year), followed by children of permanent residents (41%), naturalized parents (50%), and US-born parents (>50%) [31]. To my knowledge, no other studies have concentrated on the oral health of undocumented migrant families living in the UK, therefore, this is the first study to report findings of oral health changes in terms of parental oral health related knowledge, attitudes, and behaviors after a community-based intervention delivered to this ‘hard to reach’ population. 

Mothers in the intervention group of this study showed significant improvement in their oral health knowledge and self-reported behaviors in terms of controlling baby sugar consumption. Most of them knew that sugar was bad for teeth. It seemed that the intervention group mothers were ready to receive and assimilate this message about baby sugar snacking into their daily dietary regimens. Of interest was the finding that mothers in the intervention group reported significant improvement in their perceived importance and intervention to control baby sugar snacking. These are meaningful results as other studies have reported that prolonged bottle-feeding is found more often in migrant families [30,32]. Similar findings were reported in other parental oral health intervention programs for newly arrived migrant parents in terms of the effectiveness in improving parental oral health related knowledge, attitudes, and practices to take good care of children’s teeth [33,34]. 

The other meaningful finding was the intervention group mothers had improved knowledge, perceived importance, and intention to brush baby’s teeth, as well as their increased level of reported baby toothbrushing behaviors. These effects might be due to several reasons. First, mothers received culturally sensitive information of baby tooth decay and the importance of toothbrushing with fluoride toothpaste to prevent child tooth decay. Secondly, mothers were provided with a toothbrush and fluoride toothpaste for their infants and themselves. This meant that the financial costs and length of time adopting this healthy behavior was reduced, and thirdly, the young mothers were more responsive. It may be proposed that mothers were more ready to receive and act upon the oral health information about the welfare of their child. This was reflected in mothers being more likely to practice toothbrushing skills including establishing and assisting with child oral hygiene routines. In addition, it may be postulated that the adoption of parental toothbrushing could be thought of in terms of the developed trusting relationship with SY and adopted more “mainstream” lifestyle habits [35]. 

Despite the intervention group mothers’ improved knowledge and attitudes towards baby toothbrushing, many mothers expressed frustrations when brushing their babies’ teeth. For instance, some mothers complained that their babies would not sit quietly when having teeth brushed. These frustrations may affect mothers’ confidence to develop and maintain routine oral hygiene practice. In other words, mothers who had low self-efficacy of baby toothbrushing might be less likely to adhere to this oral hygiene regimen, as suggested by the Health Action Process Approach [36]. Similar findings were reported by Marshman and her colleagues in terms of parents’ perceived barriers to brushing children’s teeth [37]. These included parental self-efficacy of toothbrushing and their beliefs about the consequences. It further indicated the importance of social support that may exhibit as an enabling factor to empower mothers to overcome such challenges for behavior change. This is particularly significant for undocumented migrant mothers whose home based routines might be chaotic. Therefore, it may be suggested that perhaps two home visits during the intervention period are not enough for Chinese undocumented migrant mothers to develop all aspects of parenting including child oral health home care. More intensive home visits would be recommended to encourage them to develop child rearing and parenting skills in a holistic approach that incorporates baby oral health related practice as a component.

While no statistically significant changes were found in mothers’ obvious decay experience between intervention and control group mothers, this could be due to the relatively short period of the intervention to observe the changes in the clinical outcomes. The reason of including mothers’ oral health status assessment in this intervention is according to the established strong association between mothers’ active caries status and children’s caries experience [16]. Future research should consider a longer follow-up period to record changes in parental oral health status as an indicator to evaluate the effect of parental oral health education interventions.

It may be suggested that this program has shown that giving oral health education to Chinese migrant mothers in a cultural sensitive manner using a one-to-one counselling strategy can assist in raising maternal awareness of child oral health during home visits. The role of SY must be considered as a factor with regard to the effectiveness of the intervention. SY, a Chinese mandarin speaker from mainland China, conducted the program. SY shares language, culture and lifestyle habits in common with Chinese migrant mothers living in Belfast. Moreover, SY was the same age as the mothers. Therefore SY understood the Chinese migrant mothers’ psycho-social and health needs. The mothers, in turn, trusted her and consequently remained in the program. This partially explained the high retention rate in this study despite the small sample size. More importantly, this indicated the significance of the role of a community health worker sharing the same language and culture to understand and address the needs of this socially excluded group of mothers. 

### Limitation of the Study

There are several limitations in this study. The first is related to the sample. The sample gathered was a non-probability convenience sample. This is inevitable given this group is not recorded in the UK databases that could be used as sampling frames [38]. This is a most socially excluded group where the Chinese migrant population amounts to just a few thousand throughout Northern Ireland, therefore the limited sample we recruited was the entire population of accessible undocumented Chinese migrant mothers with babies aged less than eight weeks during the recruitment period. We acknowledge that the small size of the sample may increase the likelihood of type II error. Despite this concern, this work is innovative since there is little work done to promote oral health related knowledge, attitudes, and behaviors of this undocumented migrant group in Northern Ireland. 

Secondly, as SY is the researcher and also the community health worker to implement the intervention, the present study did not use any blinding measures to reduce bias, which may affect the results of the study. Further, SY’s high motivation may limit the reproductivity of similar findings for future interventions. However, this research work is regarded as meaningful for adding evidence into the limited literature to report effects of community-based interventions delivered to the undocumented migrant parents. Moreover, other studies have shown strong evidence in the effectiveness of having community health workers who share same language and culture to provide culturally appropriate health education [39]. 

Lastly, the oral health related behaviors regarding baby toothbrushing, mothers’ own toothbrushing and regular dental attendance were self-reported by mothers who might have provided socially favorable answers. This may affect the reported effectiveness of this program. Furthermore, the evaluation of the program may have been contaminated by SY. Mothers in the control group asked SY for information regarding dental health and how to access health and social services. For ethical reasons, it was impossible not to have answered their requests. This concern of contamination allows a series of questions to be raised in relation to the effectiveness of the program. It would seem reasonable to suggest the need to investigate the areas of contamination since this will assist in the development of future successful health promotion interventions for ethnic minority and migrant group parents.

## 5. Conclusions

To conclude, despite the small sample size of the present study, the community based parental oral health education program delivered in a culturally appropriate approach has shown promising effects to improve Chinese undocumented migrant mothers’ knowledge, attitudes, and self-reported behaviors with a specific emphasis on baby toothbrushing and control of baby sugar consumption. 

## Figures and Tables

**Table 1:** The procedures of the intervention program [25].

Infants’ Age	Intervention Tasks	Support Materials Provided
**8 weeks**	The community health worker is to: provide breastfeeding advice; provide weaning information; give oral health advice about baby teething, mother’s oral hygiene and regular dental attendance; encourage mothers to see a dentist and brush their teeth at least twice a day.	UNICEF Breastfeeding information leaflet; Weaning information leaflet; Baby teething ring; Mother’s toothbrush and fluoride toothpaste
**4 months**	A telephone call was made to reinforce the information given at 8 weeks	
**6 months**	The community health worker is to: emphasize the need to start to brush baby’s teeth with a smear of fluoride toothpaste as soon as first teeth erupt; demonstration of baby toothbrushing with emphasis on the smear size of fluoride toothpaste on baby; emphasize the benefits of introducing a feeding cup from 6 months onwards rather than a bottle; give advice on suitable foods and drinks for the benefits of baby’s teeth.	Baby trainer cup; Oral health pack containing baby toothbrush and fluoride toothpaste; Mother’s toothbrush and fluoride toothpaste.
**9 months**	A telephone call was made to reinforce the information given at 6 months	
**12 months**	The community health worker thanked mothers for their participation.	Baby feeding cup; Toothbrushes and fluoride toothpaste for mothers and child

**Table 2:** Demographic profiles of Chinese migrant families.

Demographic Profile	Intervention Group (*n* = 18)	Control Group (*n* = 18)	χ²	*p*-Value
Marital status				
single	1 (6%)	1 (6%)	0.00	1.00
married	17 (94%)	17 (94%)		
Number of children				
1 child	6 (33%)	9 (50%)	0.72	0.31
More than 1 child	12 (67%)	9 (50%)		
Baby gender				
female	8 (44%)	7 (39%)	0.11	0.76
male	10 (56%)	11 (61%)		
Maternal education level (number of years)				
≤12 years	11 (61%)	9 (50%)	0.45	0.50
>12 years	7 (39%)	9 (50%)		
Paternal education level (number of years)				
≤12 years	11 (61%)	8 (44%)	1.00	0.32
>12 years	7 (39%)	10 (56%)		
Social deprivation level of residential area				
Deprived area (<220)	7 (39%)	10 (56%)	1.00	0.32
Less deprived area (≥221)	11 (61%)	8 (44%)		

**Table 3:** Comparison of maternal oral health related attitudes at baseline, 6 months, and 12 months between intervention and control group mothers.

Oral Health Related Attitudes	Intervention Group Mean Scores (95% CI)	Control Group Mean Scores (95% CI)	*t*	*p*-Value
**Importance and intention to brush baby’s teeth**				
8 weeks	19.94 (19.02, 20.87)	19.61 (18.31, 20.91)	0.44	0.66
6 months	22.61 (21.56, 23.64)	18.83 (17.84, 19.83)	5.57	<0.001 ***
12 months	22.82 (21.93, 23.72)	18.76 (17.39, 20.14)	5.24	<0.001 ***
**Importance and intention to control baby sugar snacking**				
8 weeks	27.11 (25.64, 28.59)	27.94 (26.42, 29.47)	−0.83	0.41
6 months	31.11 (29.76, 32.46)	27.22 (25.91, 28.54)	4.35	<0.001 ***
12 months	32.59 (31.05, 34.13)	27.82 (26.13, 29.52)	4.40	<0.001 ***

95% CI: 95% Confidence Intervals. *** *p* < 0.001.

**Table 4:** Comparison of changes in mean scores of maternal oral health knowledge and attitudinal scales by time between intervention and control groups.

Item	Group	8 Weeks Mean (95% CI)	12 Months Mean (95% CI)	Mean Change between Groups (Baseline vs. 12 months)	*t*	*p*-Value
**Importance and intention to brush baby’s teeth**	Intervention	19.94 (19.02, 20.87)	22.82 (21.93, 23.72)	3.00 (1.56, 4.44)	3.79	0.001 **
	Control	19.61 (18.31, 20.91)	18.76 (17.39, 20.14)	−0.76 (−2.30, 0.77)		
**Importance and intention to control baby sugar snacking**	Intervention	27.11 (25.64, 28.59)	32.59 (31.05, 34.13)	5.76 (3.88, 7.65)	4.94	<0.001 ***
	Control	27.94 (26.42, 29.47)	27.82 (26.13, 29.52)	−0.18 (−1.89, 1.54)		

** *p* < 0.01, *** *p* < 0.001. 95% CI: 95% Confidence Intervals.
